# The effect of microgrooved culture substrates on calcium cycling of cardiac myocytes derived from human induced pluripotent stem cells

**DOI:** 10.1016/j.biomaterials.2012.11.055

**Published:** 2013-03

**Authors:** Christopher Rao, Themistoklis Prodromakis, Ljudmila Kolker, Umar A.R. Chaudhry, Tatiana Trantidou, Arun Sridhar, Claire Weekes, Patrizia Camelliti, Sian E. Harding, Ara Darzi, Magdi H. Yacoub, Thanos Athanasiou, Cesare M. Terracciano

**Affiliations:** a National Heart and Lung Institute, Imperial College London, United Kingdom; b Department of Surgery and Cancer, Imperial College London, United Kingdom; c Centre for Bio-Inspired Technologies, Department of Electrical and Electronic Engineering, Imperial College London, United Kingdom; d National Institute for Biological Standards and Control, South Mimms, Hertfordshire, United Kingdom; e David Jack Centre for Research and Development, GlaxoSmithKline, Ware, United Kingdom

**Keywords:** Calcium cycling, Cardiac tissue engineering, Electrophysiology, Micropatterning, Polydimethylsiloxane, Stem cells

## Abstract

Induced pluripotent stem cell-derived cardiomyocytes (iPSC-CM) have been widely proposed as *in vitro* models of myocardial physiology and disease. A significant obstacle, however, is their immature phenotype. We hypothesised that Ca^2+^ cycling of iPSC-CM is influenced by culture conditions and can be manipulated to obtain a more mature cellular behaviour. To test this hypothesis we seeded iPSC-CM onto fibronectin coated microgrooved polydimethylsiloxane (PDMS) scaffolds fabricated using photolithography, or onto unstructured PDMS membrane. After two weeks in culture, the structure and function of iPSC-CM were studied. PDMS microgrooved culture substrates brought about cellular alignment (*p* < 0.0001) and more organised sarcomere. The Ca^2+^ cycling properties of iPSC-CM cultured on these substrates were significantly altered with a shorter time to peak amplitude (*p* = 0.0002 at 1 Hz), and more organised sarcoplasmic reticulum (SR) Ca^2+^ release in response to caffeine (*p* < 0.0001), suggesting improved SR Ca^2+^ cycling. These changes were not associated with modifications in gene expression. Whilst structured tissue culture may make iPSC-CM more representative of adult myocardium, further construct development and characterisation is required to optimise iPSC-CM as a model of adult myocardium.

## Abbreviations

α-MHC: alpha-myosin heavy chain
β-MHC: beta-myosin heavy chain
CALR: calreticulin
CASQ2: calsequestrin 2
CAV3: caveolin 3
Cav3.1: T-type Ca^2+^ channel
Cav1.2: L-type Ca^2+^ channel
cTNT: cardiac troponin T
Cx43: connexin 43
GAPDH: glyceraldehyde-3-phosphate dehydrogenase
IP3R: inositol trisphosphate receptor
iPSC: induced pluripotent stem cell
iPSC-CM: induced pluripotent stem cell-derived cardiomyocytes
JPH2: junctophilin 2
MLC2V: myosin light chain 2v
NCX: sodium–calcium exchanger
NT: normal Tyrode's solution
PDMS: polydimethylsiloxane
PLN: phospholamban
RyR: ryanodine receptor
SERCA2a: sarcoplasmic reticulum Ca^2+^ ATPase
SR: sarcoplasmic reticulum
TRDN: triadin
UV: ultraviolet

## 1 Introduction

Induced pluripotent stem cells (iPSC) are generated by forced expression of embryonic transcription factors and have several features that make them ideally suited to study myocardial pathology and function. They can be cultured for many months without loss of normal karyotype, transfected, and can be readily differentiated into cardiomyocytes [1]. iPSC-derived cardiomyocytes (iPSC-CM) have been used to replicate the phenotypes of several inherited cardiac diseases *in vitro* [2–5]. Significantly they have also been used to suggest novel therapies [5] and molecular mechanisms that may underlie pathological processes [4]. Finally, iPSC-CM have widely been proposed as a screening tool for toxicology [1,6].

There is evidence however, that iPSC-CM have gene expression characteristics of developing cardiomyocytes [7], immature ultrastructural phenotypes [8,9], immature electrophysiological properties [10] and abnormal Ca^2+^ cycling [11]. Furthermore, iPSC-CM exhibit a heterogeneous phenotype, for example, usually representing a mixed population of cells with the diverse electrophysiological characteristics of nodal, atrial and ventricular cells [12]. Whilst it may be possible to use iPSC-CM to study single ion channel disorders, or inherited cardiomyopathies with a catastrophic cellular and molecular phenotype that present early in childhood, their immature phenotype excludes their application to complex pathologies and cardiomyopathies with an acquired component. In particular, the lack of mature Ca^2+^ cycling properties in iPSC-CM appear to be an important obstacle, as Ca^2+^ cycling plays a critical role in the cellular phenotype of both inherited [13] and acquired cardiomyopathies [14].

Structured tissue culture substrates that bring about regular alignment and anisotropy on the cell culture have been previously used to improve Ca^2+^ cycling properties and sarcomeric organisation of neonatal rat ventricular myocytes (NRVM) [15–20]. Several different approaches have been employed, including using substrates coated in micropatterned extracellular matrix components such as fibronectin [15,16], microgrooved load [17] and nanogrooved culture substrates [18], and more sophisticated three-dimensional constructs [19,20]. However, it is not clear whether these strategies are effective when using iPSC-CM.

In this study we hypothesise that Ca^2+^ cycling of iPSC-CM is influenced by structured culture substrates and can be manipulated to obtain a more mature cellular behaviour. To test this hypothesis we cultured iPSC-CM on polydimethylsiloxane (PDMS) microgrooved substrates. This approach is a simple, cost-effective and reproducible technique. It also has proven efficacy in NRVM where it has been shown to promote cell alignment and to increase the speed and amplitude of calcium cycling [17,21]. In addition to Ca^2+^ transients and SR Ca^2+^ regulation, we studied action potential properties, nuclear alignment, sarcomeric organisation, and gene expression to investigate the effects that structured culture substrates have on Ca^2+^ cycling and the underlying mechanisms.

## 2 Methods

### 2.1 Fabrication of microgrooved PDMS culture constructs

**Fig. 1:**
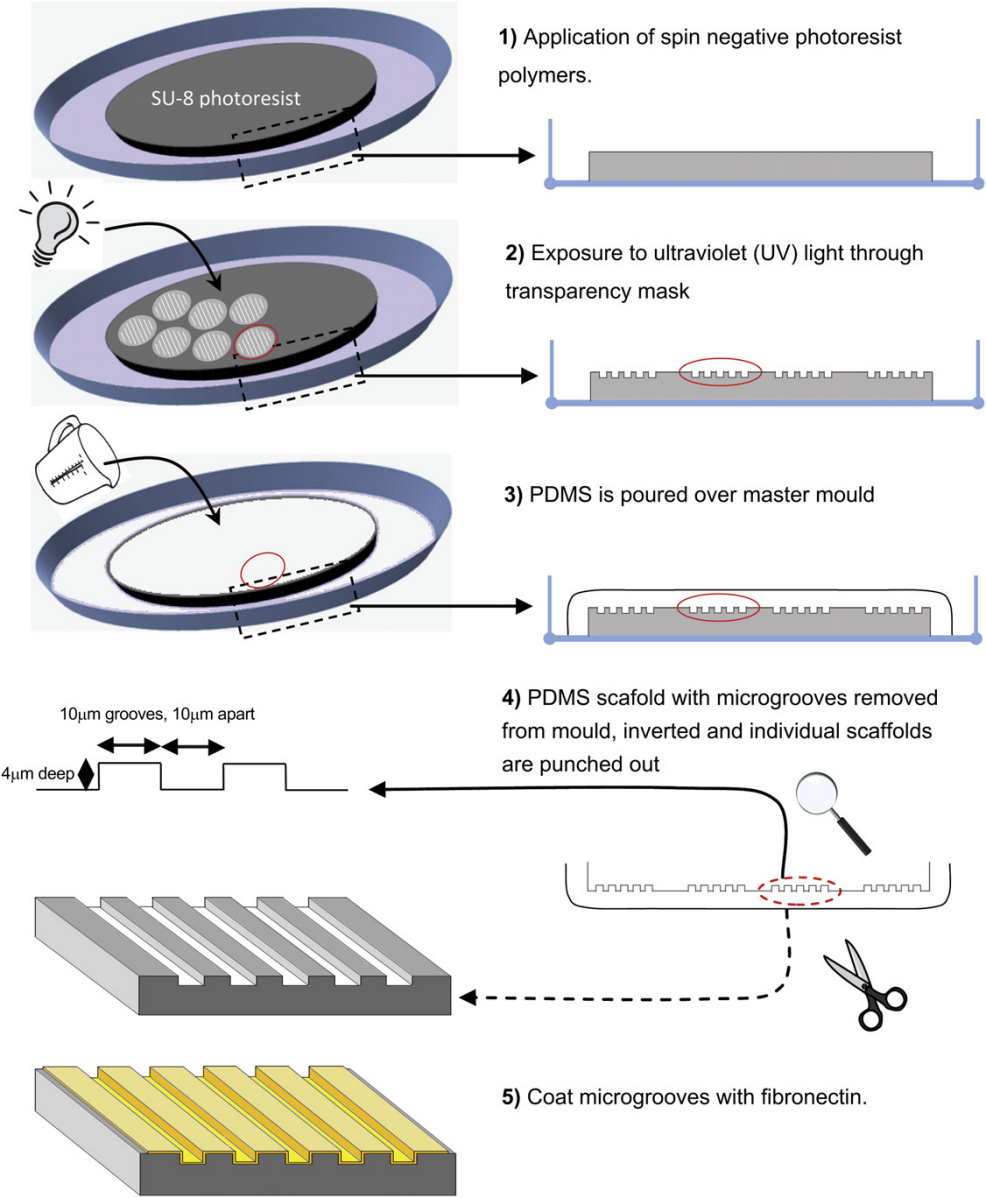
Schematic demonstrating the fabrication of microgrooved tissue culture substrates (not drawn to scale).

Structured microgrooved flexible tissue culture substrates were fabricated from PDMS, a biologically inert non-toxic polymer [22] via standard photolithography rules, as previously described [23]. Briefly, SU-8 photoresist polymer was spun onto monocrystalline silicon wafers. The photoresist was then exposed to ultraviolet (UV) light (365 nm) through a chrome-plated glass mask, comprising transparent areas with parallel grooves and developed using 100% ethyl lactate. The resulting SU-8 mould had patterned circular areas 14 mm in diameter, with parallel lines etched into them 10 μm apart, 10 μm wide and 4 μm deep. The dimensions were chosen as preliminary studies with NRVM suggested that microgrooves of this width most effectively aligned and modified the Ca^2+^ cycling properties of cardiomyocytes (Supplemental Data, Figs. 1 and 2). This was also supported by other previous studies [24]. PDMS was prepared by mixing a pre-polymer and a curing agent (Sylgard 184 Kit; Dow Corning). It was then casted onto the SU-8 mould, was thoroughly de-gassed in vacuum and was cured at 70 °C for 1 h before being carefully cut from the master mould. The individual circular patterned areas on the constructs were then removed using a 3/4 inch carpentry punch to fit into the bottom of a 12-well plate and a 9/16 inch carpentry punch to fit into the bottom of a 24-well plate. The larger constructs were used for electrophysiological applications and the smaller constructs were used for imaging and molecular biological applications. Unstructured tissue culture constructs were fabricated on the flat surfaces of the membranes by simply inverting the microgrooved PDMS substrates. This ensured that the structured and unstructured constructs had similar mechanical properties and stiffness. The constructs were then sterilised by emersion in 70% ethanol and 4 h expose to UV light. The following day the constructs were rinsed three times with sterile water, coated with 50 μg/ml human-plasma fibronectin (Sigma–Aldrich) and then left for at least 4 h prior to plating. Excess fibronectin was removed immediately prior to plating of the cells. 1/3 million iPSC-CM, iCell Cardiomyocytes™ (Cellular Dynamics International, Wisconsin USA) were seeded in each well in the 12-well-pate. 1/6 million iCell cardiomyocytes™ were seeded in each well in the 24-well-pate. iPSC-CM were seeded and maintained according to manufactures guidelines. All calcium studies, structural, and gene expression studies were performed 2 weeks following seeding of iPSC-CM (Fig. 1).

### 2.2 Immunohistochemistry

Constructs were fixed in 4% paraformaldehyde in 0.1 m phosphate buffer (Agar Scientific) for 10 min, washed in PBS (Sigma–Aldrich) and permeabilized using 0.2% triton-X (Sigma–Aldrich) in PBS for 3 min followed by two PBS washes. The coverslips were then incubated with blocking solution containing 3% BSA (Sigma–Aldrich) in PBS for 30 min. Various primary antibodies (Mouse α-Actinin IgG Ascites, 1:100, Sigma–Aldrich; Mouse Ryanodine Receptor (RyR) IgG 1 mg/ml, 1:500, Abcam; Rabbit Ca_v_1.2 IgG 0.8 mg/ml, 1:100, Alomone Labs; Mouse Phospholamban (PLN) IgG 1 mg/ml, 1:200, Badrilla; Rabbit Connexin 43 (Cx43) IgG 0.5 mg/ml, 1:50, Millipore) were added for 1 h at room temperature. The constructs were then washed at least 3 times in PBS for 3–5 min each. Secondary antibodies were then added (Alexa Fluor 488 anti-mouse, Alexa Fluor 488 anti-rabbit, Alexa Fluor 555 anti-mouse, and Alexa Fluor 555 anti-rabbit; all goat IgG, 2 mg/ml, 1:800; Invitrogen) and incubated for 1 h at room temperature. The constructs were then washed again at least 3 times in PBS for 3–5 min each. This was repeated for each subsequent label. Finally constructs were washed twice with 300 nm DAPI (Invitrogen) in PBS for 3–5 min each. Fluorescence imaging was performed using LSM510 confocal microscope using a ×40 oil-immersion lens.

### 2.3 Assessment of alignment

iPSC-CM alignment was quantified using DAPI images which were converted into binary images using ImageJ. The long axis of each nucleus was measured relative to the horizontal axis of the image field using NIS-Elements AR3.2 software (Laboratory Imaging, Nikon). Objects were gated according to size exclude non-nucleus or composite structures. Alignment was defined as the lack of deviation in the axis of individual nucleus from the mean axis of all individual nuclei. In order to quantify iPSC-CM alignment the mean axis was first calculated, and then the variance of the minimum angle between the long axis of each nucleus and the mean axis of all nuclei was compared using an F-test of equality of variances. The mean angle between the long axis of each nucleus and the mean axis of all nuclei was calculated. Analysis of colocalisation was performed using the WCIF ImageJ plugin bundle (Wright Cell Imaging Facility, Toronto Research Institute).

### 2.4 Action potential measurement

As previously described [25], action potential (AP) measurements were performed using an Axoclamp 2B system (Axon Instruments). High resistance microelectrodes were used (15–25 MΩ) (Harvard Apparatus). Cells were superfused with 37 °C Normal Tyrode's (NT) solution containing; 140 mm NaCl, 6 mm KCl, 1 mm MgCl_2_, 1 mm CaCl_2_, 10 mm glucose, 10 mm HEPES adjusted to pH 7.4 with 2 m NaOH (All Sigma–Aldrich); and the microelectrode filling solution contained; 2 m KCl, 0.1 mm EGTA, 5 mm HEPES adjusted to pH 7.2 with 2 m NaOH (All Sigma–Aldrich). Action potentials were recorded in current clamp mode and measured AP were analysed using pCLAMP 10.3 software (Molecular Devices).

### 2.5 Measurement of Ca^2+^ transients

iPSC-CM were loaded with 20 μm Fluo-4 acetoxymethyl ester (Invitrogen) using 8 μl (250 nm) probenecid (Invitrogen) and 0.2% pluronic acid (Invitrogen), in 2 ml pre-warmed DMEM (Invitrogen) at 37 °C for 30 min. The myocytes were then washed and incubated with pre-warmed DMEM containing 2% FBS (Invitrogen) and 250 nm probenecid for 30 min to de-esterify. The experimental dish was mounted on the stage of an upright Zeiss LSM510 confocal microscope (Carl Zeiss) and myocytes were observed through a ×40 water immersion objective. Line scanning was performed at suitable regions with the myocytes spontaneously beating or under field stimulation at 0.5 Hz, 1 Hz using an external pacing generator. During recording the cells were superfused with 37 °C NT or Na^+^ and Ca^2+^ free solution containing: 140 mm LiCl, 6 mm KOH, 1 mm MgCl_2_, 10 mm glucose, 10 mm HEPES, 0.1 mm EGTA adjusted to pH 7.4 with 2 M NaOH (All Sigma–Aldrich). 50 mm caffeine (Sigma–Aldrich) was used for sarcoplasmic reticulum (SR) studies. Linear time–length images were converted into Ca^2+^ transients using ImageJ (National Institutes of Health) and analysed using pCLAMP 10.3. Fluorescent values were normalised to baseline fluorescence (f/f0). tP was taken as the time taken for the ratio signal to reach peak fluorescence from baseline fluorescence. Similarly, t50 and t90 were taken as the time taken for the fluorescent transient to decline by 50% and 90% of the transient amplitude respectively [26].

### 2.6 Gene expression

Total RNA from iPSC-CM was isolated using the RNeasy Mini Kit (Qiagen). Genomic DNA was removed by DNase I (Invitrogen) treatment and total RNA (500 ng) was reverse transcribed into cDNA. qPCR was performed using 150 ng of cDNA using SensiMix SYBR No-ROX Kit (Bioline, UK) on the Rotor-Gene™ 6000 (Corbett Research). Primers were designed using the Universal Probe Library (UPL) (Roche) (Table 1). Gene expression levels in iPSC-CM were compared to total RNA was isolated from a human fibroblast line [27], and to commercially available adult human (Agilent) and foetal human heart total RNA (Agilent). All values were normalised to Glyceraldehyde-3-phosphate dehydrogenase (GAPDH) expression and expressed relative to gene expression in the adult heart.

### 2.7 Statistical analysis

Statistical analysis was performed using a Fisher exact test, unpaired Mann–Whitney *U* test or 1-way ANOVA Kruskall–Wallis test where appropriate. Dunn's post-hoc test was used to test for differences between groups. Data are expressed as mean ± SEM unless specified otherwise. For Ca^2+^ cycling studies and AP measurements, *n* represents the number of myocytes. For gene expression studies *n* represents the number of biological replicates. In the figures, *indicates *p* < 0.05; **p < 0.01; and ***p < 0.001. The analysis was performed using Prism 4 software (GraphPad software Inc).

## 3 Results

### 3.1 Cell alignment and sarcomere structure

**Fig. 2:**
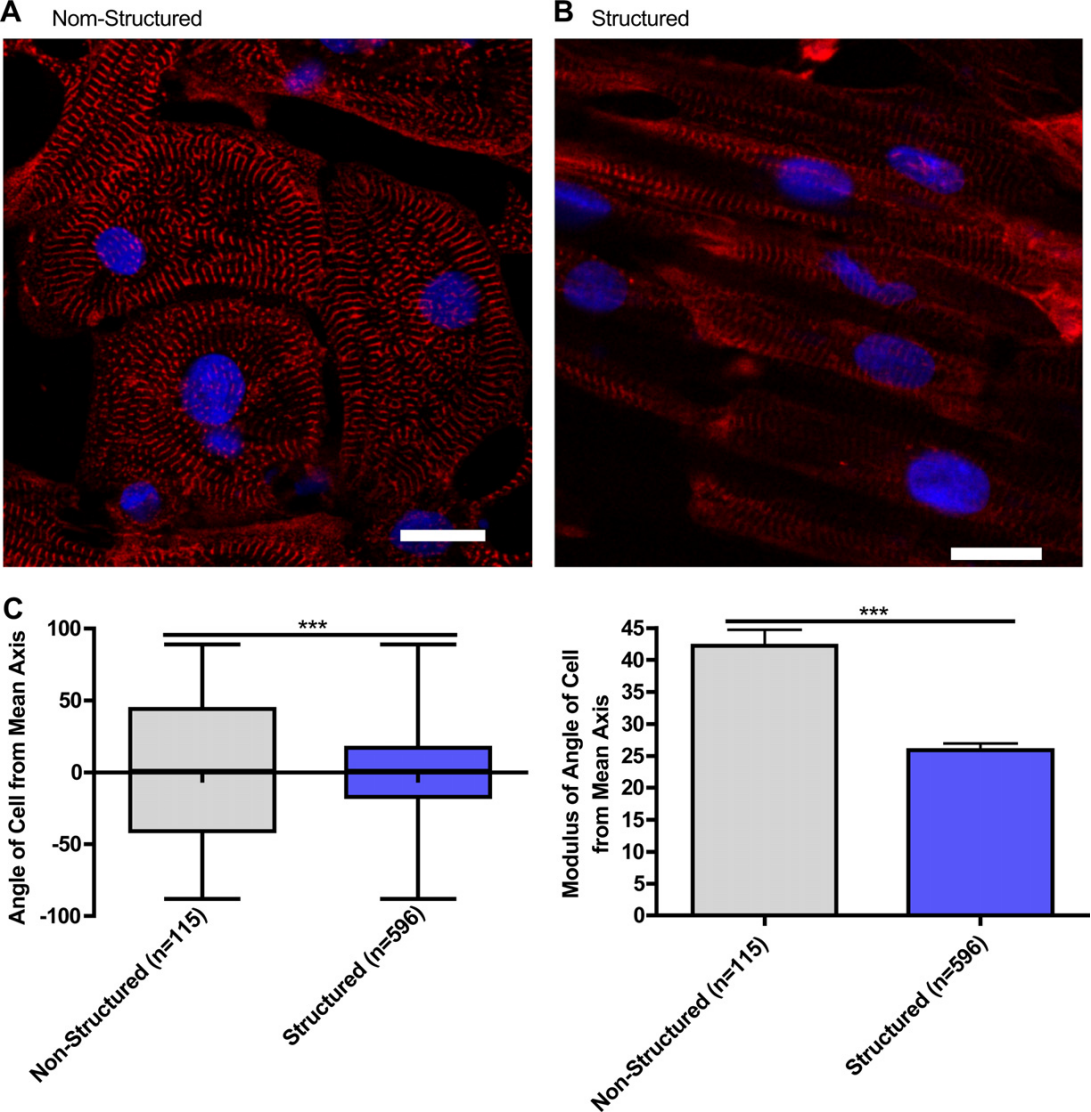
Representative immunofluorescence of iPSC-CM cultured on unstructured PDMS (A) and microgrooved PDMS (B), Red – sarcomeric α-actin, Blue – DAPI, scale bar 20 μm. Quantification of cell alignment iPSC-CM on structured and unstructured constructs (C). (For interpretation of the references to colour in this figure legend, the reader is referred to the web version of this article.)

Microgrooved PDMS substrates significantly improved iPSC-CM alignment compared to the unstructured substrates (SD of Unstructured 50.11° *n* = 115, Structured 35.60° *n* = 596; *F* = 1.982, *p* < 0.0001). This resulted in more organised sarcomeric structures as seen in the aligned α-actinin striation pattern of the myofibrils (Fig. 2).

### 3.2 Calcium cycling

**Fig. 3:**
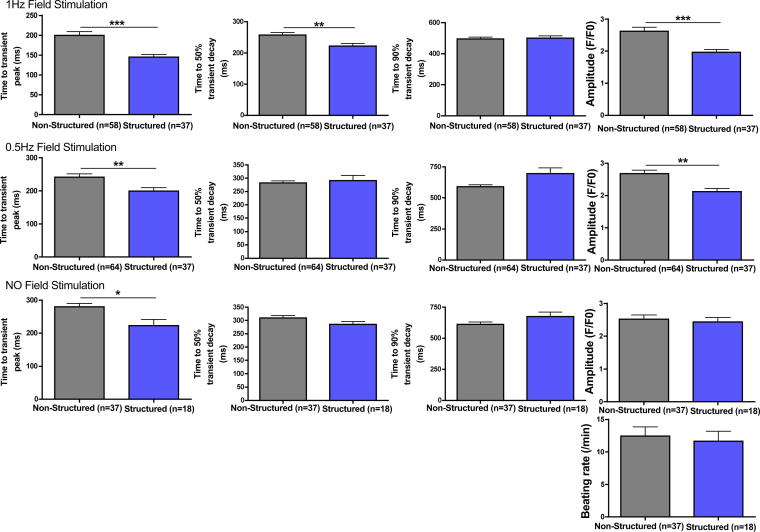
Time to peak of the Ca^2+^ transient (tP), 50% decay (t50), 90% decay (t90), and fluorescence amplitude (fp/f0) of iPSC-CM cultured on unstructured PDMS and microgrooved constructs field-stimulated at 1 Hz, 0.5 Hz, and beating spontaneously.

**Fig. 4:**
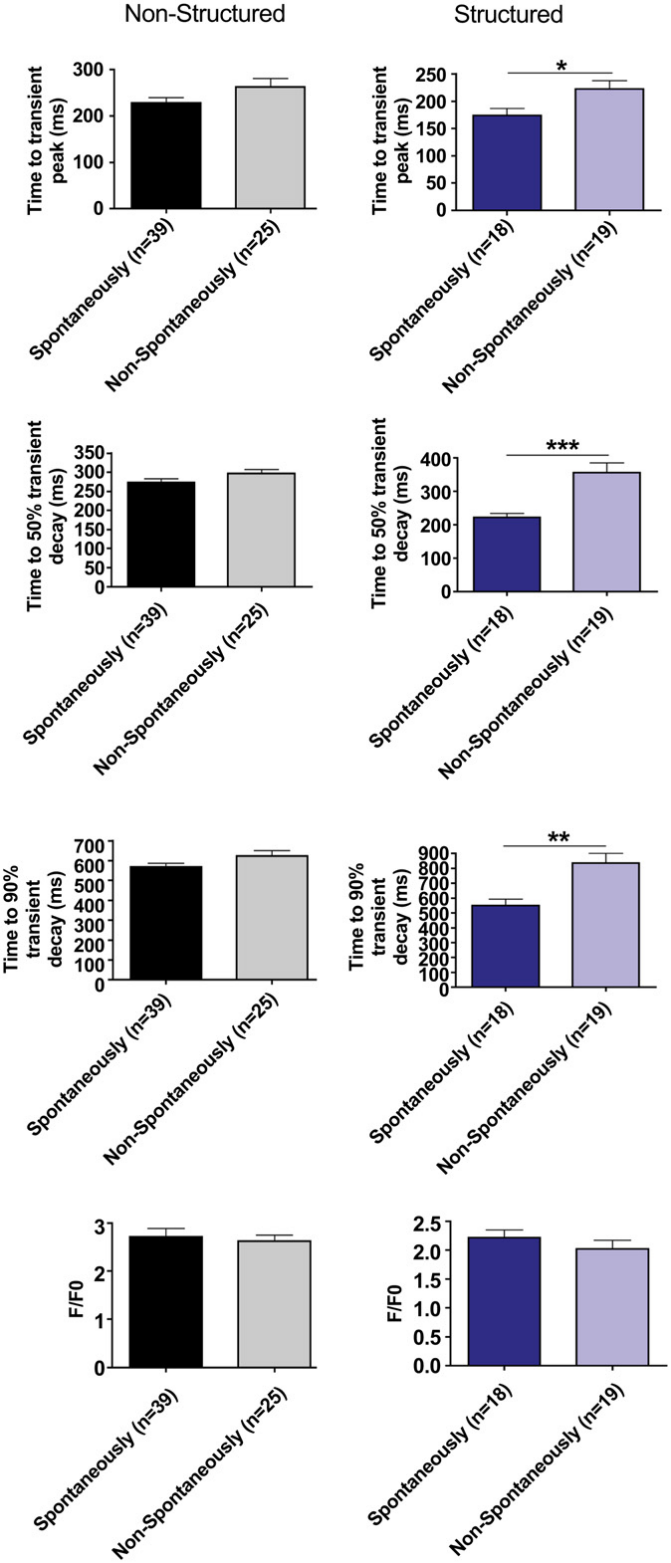
Time to peak of the Ca^2+^ transient (tP), 50% decay (t50), 90% decay (t90), and fluorescence amplitude (fp/f0) of spontaneously beating and non-spontaneously beating iPSC-CM cultured on structured and control substrates field-stimulated at 0.5 Hz.

iPSC-CM cultured on structured substrates had a shorter time to peak Ca^2+^ transient amplitude (tP) when stimulated at 1 Hz (*p* = 0.0002) and time to 50% transient decay (t50) (*p* = 0.0065). There was no change in the time to 90% decay (t90). At 0.5 Hz there was a shorter tP (*p* = 0.0073) but no changes in t50 or t90. Similarly while iPSC-CM were beating spontaneously, there was a reduced tP (*p* = 0.0012) in structured cells but no change in the t50 or t90. At 1 Hz (*p* = 0.0004) and 0.5 Hz (*p* = 0.0023) the amplitude was significantly reduced in the iPSC-CM cultured on microgrooved PDMS substrates, however not when beating spontaneously. There was no significant difference in the rate of spontaneous Ca^2+^ transients (Structured: 11.67 beats per minute ±1.495, *n* = 18; Unstructured: 12.43 beats per minute ±1.432, *n* = 37; *p* = 0.8859) (Fig. 3). Similarly the proportion of iPSC-CM with spontaneous Ca^2+^ transients did not differ significantly between groups (Structured: 18/37 (48.6%); Unstructured: 37/64 (57.8%); *p* = 0.73). iPSC-CM spontaneously beating on structured tissue culture substrates had significantly reduced tP, t50 and t90 when field-stimulated at 0.5 Hz compared with cells without spontaneous activity in culture, however this difference was not seen in unstructured constructs (Fig. 4).

**Fig. 5:**
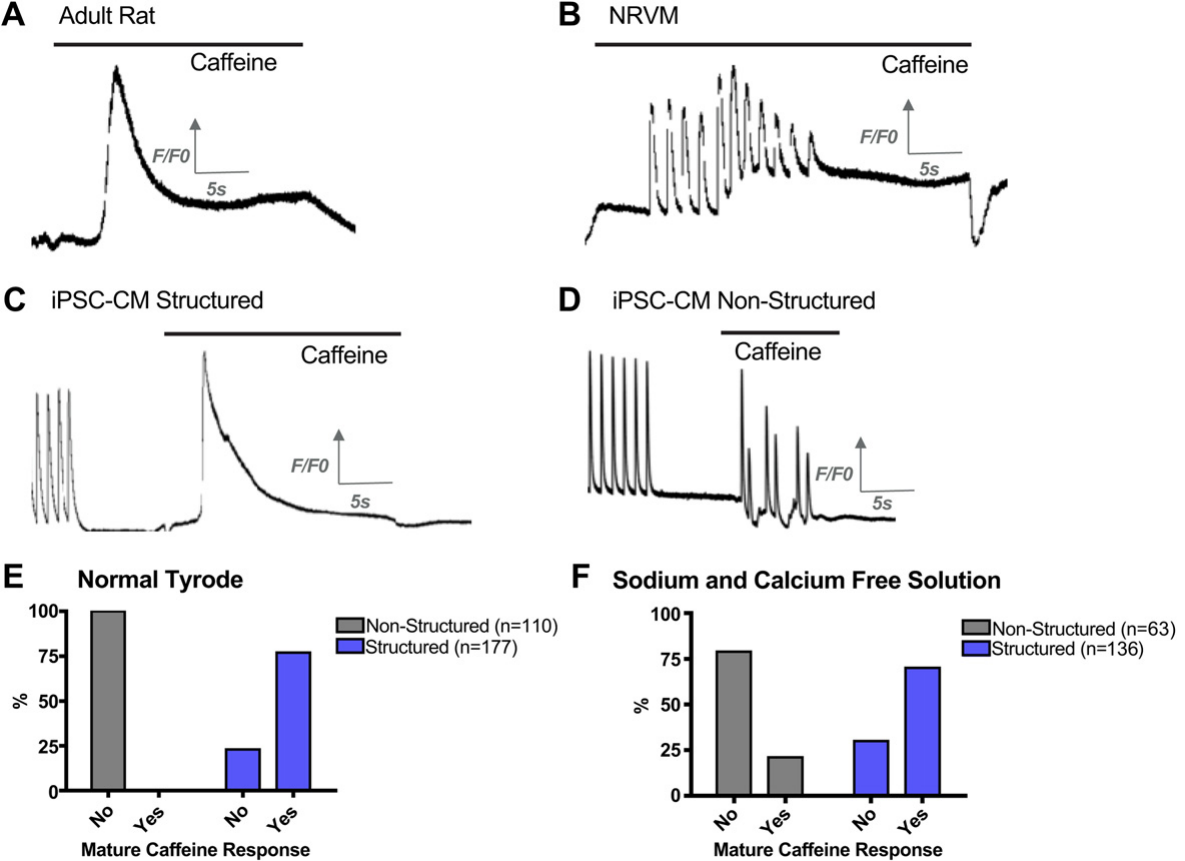
Representative traces showing response to the application of 50 mm caffeine solution of isolated adult rat ventricular cells illustrating “mature caffeine response” (A), NRVM illustrating “immature caffeine response” (B), iPSC-CM cultured on structured PDMS (C), and iPSC-CM cultured on unstructured PDMS (D). Proportion of experiments that elicited an organised response to caffeine when cells were superfused in NT (E). Proportion of experiments that elicited an organised response to caffeine when cells were superfused in Na^+^ and Ca^2+^ free solution (F).

In order to investigate whether differences in tP between structured and unstructured cells were due to differences in SR Ca^2+^ release, iPSC-CM were spritzed with solutions containing high concentrations of caffeine, as previously described [9,11]. A “synchronous” SR Ca^2+^ release was elicited in response to caffeine containing NT in 77% of the structured iPSC-CM consisting of a single large transient. However, with iPSC-CM cultured on unstructured constructs we observed multiple peaks of the caffeine-transient indicating irregular, asynchronous release from the SR (*p* < 0.0001) (Fig. 5). The experiments were repeated in Na^+^ and Ca^2+^ free solution to exclude extracellular calcium cycling by preventing Ca^2+^ extrusion via the sodium–calcium exchanger (NCX), or L-type Ca^2+^ current-mediated Ca^2+^ induced Ca^2+^ release. Again, “synchronous” SR Ca^2+^ release was observed in 70% of structured constructs but in only 21% of unstructured constructs (*p* < 0.0001) suggesting that this effect was independent on sarcolemmal fluxes. Overall our data suggest that SR Ca^2+^ regulation is improved by culture on microgrooved PDMS substrates (Fig. 5).

### 3.3 Action potential duration

**Fig. 6:**
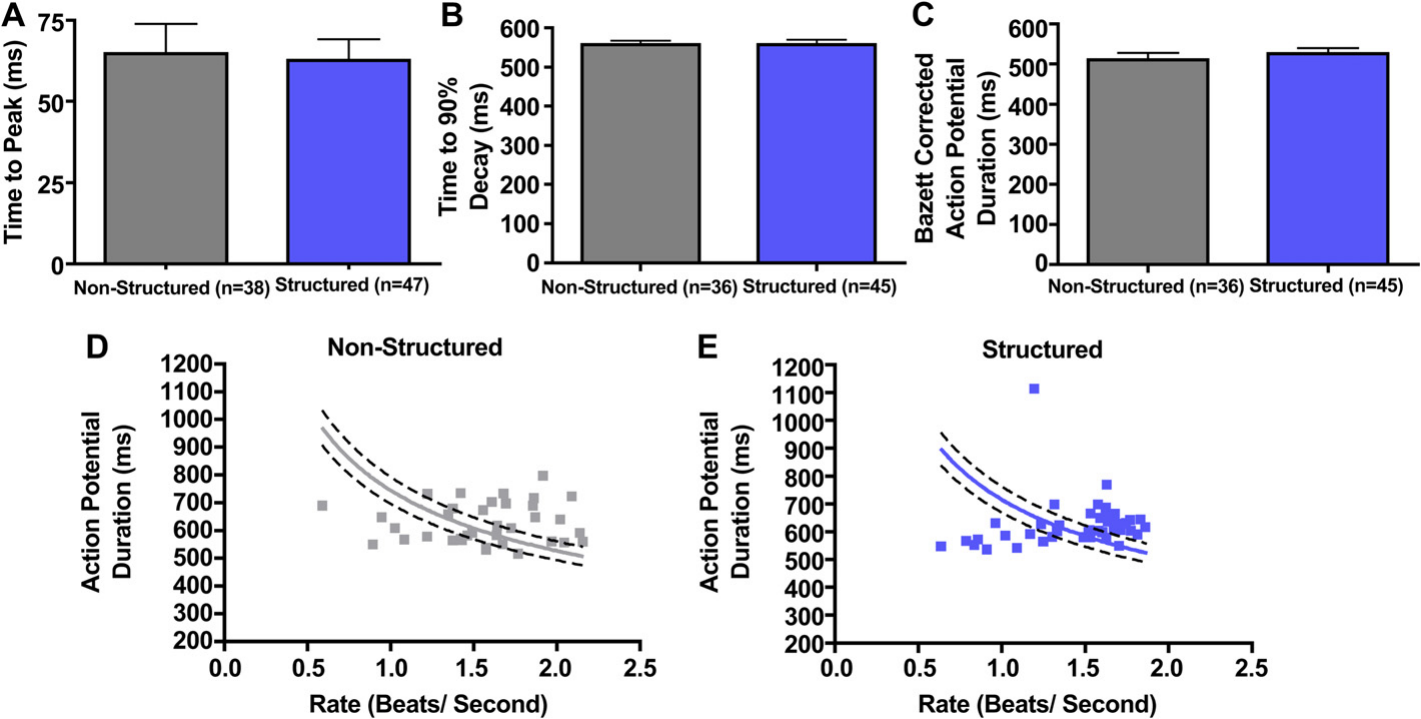
Spontaneous APD measured using sharp microelectrodes (A), spontaneous beating rate (B), and APD corrected for spontaneous beating rate (C). Panels D and E suggest that Bazett's correction (curved line) does not adequately describe the relationship between APD and beating rate.

There was no significant difference in the spontaneous AP rate in either group (*p* = 0.16) (Fig. 6). Both the uncorrected (*p* = 0.8904) and Bazett's corrected APD (*p* = 0.46) were not significantly different, however in both groups the rate–APD relationship was not well described by the Bazett's formula (Fig. 6).

### 3.4 Protein localisation

**Fig. 7:**
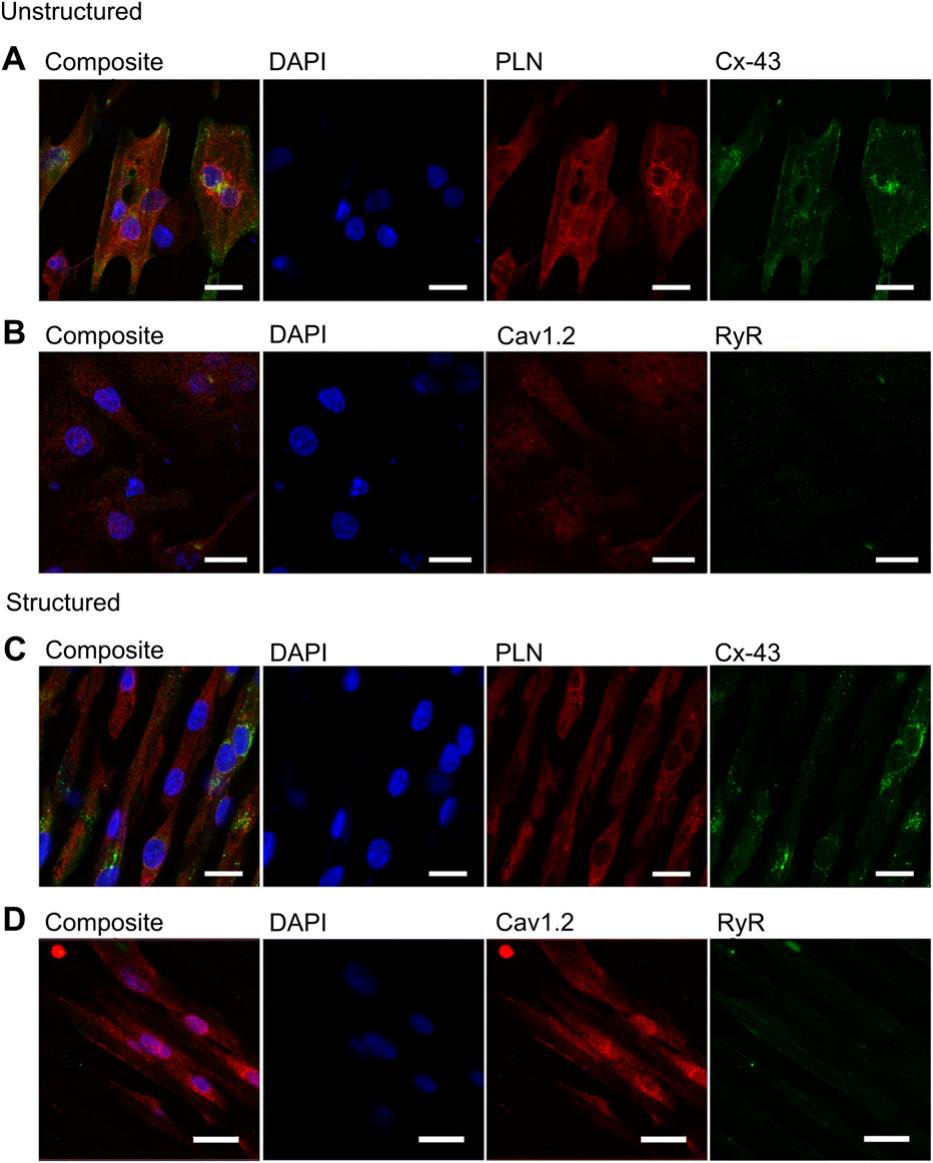
(A) Immunohistochemistry of iPSC-CM cultured non-structured PDMS, Red – PLN, Green – Cx43, Blue – DAPI, scale bar 20 μm. (B) Red – Cav1.2 channel, Green – RyR, Blue – DAPI, scale bar 20 μm. (C) Immunohistochemistry of iPSC-CM cultured structured PDMS, Red – PLN, Green – Cx43, Blue – DAPI, scale bar 20 μm. (D) Red – Cav1.2 channel, Green – RyR, Blue – DAPI, scale bar 20 μm. (For interpretation of the references to colour in this figure legend, the reader is referred to the web version of this article.)

We did not find evidence that other ultrastructural properties were affected by alignment of iPSC-CM on microgrooved PDMS substrates. For example, Cx43 did not appear to be preferentially expressed along the short axis of aligned cells, as in adult cardiomyocytes, and RyR and PLN expression did not suggest that SR organisation was improved in structured iPSC-CM. Notably, the cells showed only week staining for RyR suggesting a low expression of the receptor, which is confirmed by qPCR (Fig. 7). Colocalisation of RyR and Ca_v_1.2 was increased in the structured group (Structured: Pearson's coefficient (*r*) = 0.028, *n* = 6 images; Unstructured: *r* = −0.183, *n* = 4 images; *p* < 0.001). However this must be interpreted with caution given the minimal area colocalized in both groups (0.08% of all image pixels in the structured group compared to 0.24% in the unstructured group).

### 3.5 Gene expression

**Fig. 8:**
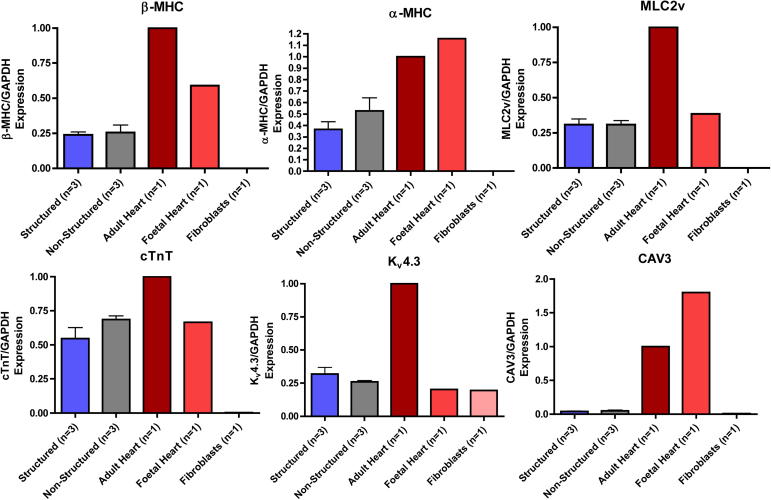
Comparison of expression of genes encoding ultrastructural proteins in cardiomyocytes (α-MHC, β-MHC, MLC2V, cTNT, BIN1, CAV3) when normalised to GAPDH and expressed relative to adult heart tissue in iPSC-CM cultured on structured and control substrates, fibroblasts, adult heart and foetal heart tissue.

**Fig. 9:**
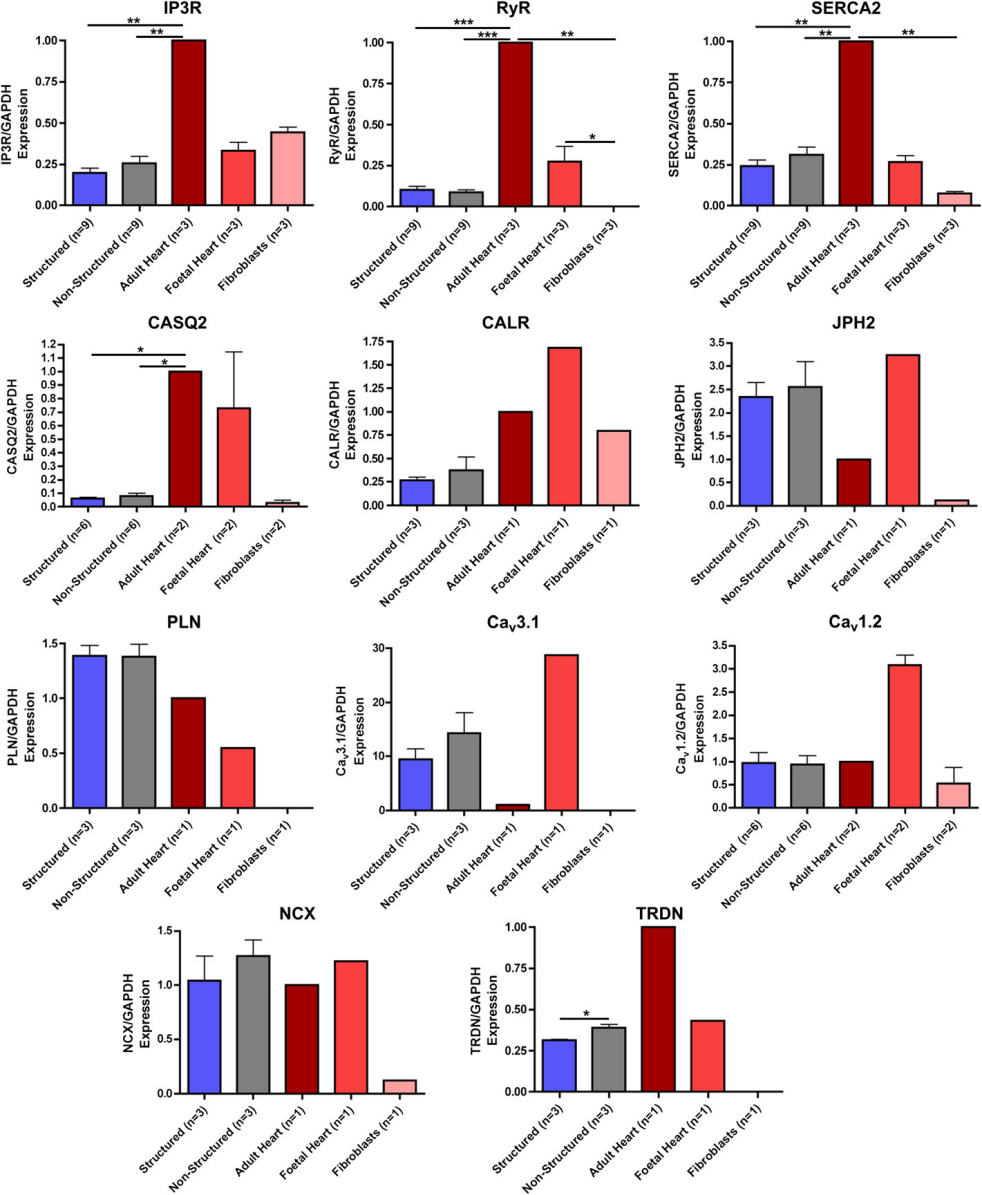
Comparison of expression of genes encoding proteins important for Ca^2+^ cycling in cardiomyocytes (IP3R, RyR, SERCA2a, CASQ2, CALR, JPH2, PLN, Ca_v_3.1, Ca_v_1.2, NCX and TRDN) when normalised to GAPDH and expressed relative to adult heart tissue in iPSC-CM cultured on structured and control substrate, fibroblasts, adult heart and foetal heart tissue.

The expression patterns of genes encoding structural proteins such as alpha-myosin heavy chain (α-MHC), beta-myosin heavy chain (β-MHC), myosin light chain 2v (MLC2V), cardiac troponin T (cTNT), caveolin 3 (CAV3) (Fig. 8) and those important for Ca^2+^ cycling (inositol trisphosphate receptor (IP3R), RyR, sarcoplasmic reticulum Ca^2+^ ATPase (SERCA2a), calsequestrin 2 (CASQ2), calreticulin (CALR), junctophilin 2 (JPH2), PLN, T-type Ca^2+^ channel (Cav3.1), L-type Ca^2+^ channel (Cav1.2), NCX and triadin (TRDN)) (Fig. 9) were similar in structured and unstructured iPSC-CM, and equally different from adult myocardium with gene expression levels generally close or below foetal heart controls. There was no significant difference in the expression of any gene except triadin (*p* = 0.0250). Gene expression of early cardiac transcription factors and genes associated with pluripotency was higher in iPSC-CM, however there was no difference between structured and unstructured constructs (Supplementary Fig. 3). For normalisation of gene expression data, GAPDH was employed but the use of alternative house-keeping genes such as 18s ribosomal RNA, Cyclophilin G, and β-actin did not change the results of our analysis (Supplementary Fig. 4).

## 4 Discussion

iPSC-CM cultured on microgrooved PDMS substrates adopted structural properties such as cellular alignment and sarcomeric organisation which resembled adult cardiomyocytes. iPSC-CM on microgrooved PDMS substrates also had shorter tP and t50 when stimulated at 1 Hz. When stimulated at 0.5 Hz, and when spontaneously beating, structured iPSC-CM also had a shorter tP. The spontaneous beating rate and action potential duration was unchanged between groups. More organised SR Ca^2+^ release was elicited in response to caffeine in structured iPSC-CM.

The finding that structured tissue culture substrates promote alignment of iPSC-CM and improve sarcomeric organisation is supported by several studies in the literature in which NRVM have been aligned in an anisotropic fashion using a variety of physical external stimuli including micro [21] and nanogrooves [18], substrate stiffness [28] and, patterning of extracellular matrix components [29]. All these methods appear to promote homogeneously aligned cells, elongated along the axis of alignment with a smaller minor axis [15,21,24]. Alignment of myofibrillar, cytoskeletal and sarcomeric structures is widely reported in the literature and is constant with our findings in human iPSC-CM [18,24,28,29]. It has been suggested that anisotropic focal adhesion complexes form parallel to the grooves [28], and this, together with evidence on the strain exerted on the substrate at a sub cellular level [20], implies that the load that the cells exert on themselves may be an important factor in the development elongated cells with aligned myofibrillar, cytoskeletal and sarcomeric structures. There is also evidence to suggest that nuclear morphology is also altered. Cell alignment with external stimuli appears to promote binucleation, and higher nuclear eccentricity such as in adult cardiomyocytes [24,30]. We did not find any evidence for an increase in binucleation, however the nuclei in the structured group were more elliptical.

Several studies report that aligned cells express more Cx43 in clusters [21] localised in a bipolar fashion analogous to adult cells [31], and have higher conduction velocities in the longitudinal direction [18,21]. We did not find a marked difference in the distribution of Cx43, and we did not investigate conduction velocity anisotropy. Like other groups we also did not find a difference between the action potential morphology of structured and unstructured cells [16] and whilst higher synchronous beating rates have been reported in anisotropic cultures [28] and increased maximum capture rate in response to electrical stimulation in structured culture have also been described [16], we did not find any statistically significant difference in the spontaneous beating rate of the structured and unstructured group.

We found that structured tissue culture substrates significantly changed the Ca^2+^ cycling properties of iPSC-CM, reducing the tP. This could be due to changes in Ca^2+^ entry and trigger for CICR. Immunohistochemistry suggests that Ca_v_1.2 and RyR were poorly colocalized and the slight improvement in the structured cultures is unlikely to explain the faster tP. Although expression of Ca_v_1.2 was not significantly different between groups it is possible that post translational modification may result in differential expression of Ca_v_1.2 at the sarcolemma and extracellular Ca^2+^ influx may explain the faster tP.

Similarly, changes in intracellular Ca^2+^ buffering may also account for the changes in Ca^2+^ cycling induced by microgrooved PDMS substrates. The marked difference between the responses of iPSC-CM to caffeine suggests that the observed differences may also be partly due to regulation of Ca^2+^ by the SR. Ca^2+^ release from the SR is predominantly mediated by the RyR in adults, although in immature cardiomyocytes the IP3R plays a more significant role [32]. A difference in the ratio of the RyR and IP3R receptors, or difference in the absolute number of either receptor may explain our findings. The gene expression data presented here does not support this hypothesis. Phosphorylation of the RyR, or other factors including SR Ca^2+^ content, which is known to increase the open probability, may also explain these findings [33]. Given that in the unstructured group, several irregular Ca^2+^ transients were observed upon application of caffeine, SR Ca^2+^ could not be quantified. The finding that triadin was more highly expressed in unstructured cells compared to structured cells is interesting as triadin overexpression has been shown to block excitation–contraction coupling in myotubes and cardiomyocytes in the absence of extracellular Ca^2+^ [34]. However, the marginal raise in triadin, alone, is unlikely to explain the differences in Ca^2+^ cycling that we observed; firstly, despite being significantly raised in iPSC-CM cultured on unstructured tissue culture substrates; it falls well below the levels seen in adult cardiomyocytes. Secondly, this change would not be expected to have an effect on caffeine induced transients, and finally it is unclear from the literature what effect a small increase in triadin would have in the presence of extracellular calcium, especially given the multiple isoforms, all with potentially different functions [34,35]. More studies are required to determine the role of SR in the Ca^2+^ cycling effects observed in the microgrooved PDMS cultures.

Whilst the role of structured tissue culture substrates has not previously been studied in iPSC-CM, their effect on Ca^2+^ cycling has been studied in NRVM. Several studies suggest that structured constructs have lower diastolic Ca^2+^ levels. Structured substrates also have been shown to reduce diastolic Ca^2+^ levels in several [15,16,24] but not all studies [21]. It has also been suggested that elongation using aligned collagen constructs increases voltage-gated Ca^2+^ currents and alters their regulatory properties [36]. In contradiction to our findings several studies report an increase in the amplitude of Ca^2+^ transients [17,21,24] or systolic Ca^2+^ levels [21], similarly many studies report increased SR Ca^2+^ content [17,21]. Whilst our study did, like several studies, show faster calcium transient peak, we did not see any effect on Ca^2+^ extrusion [15,16,24]. The implication is that whilst Ca^2+^ release mechanisms from the SR have become more representative of adult myocardium, Ca^2+^ uptake mechanisms have not undergone a similar change. This is supported by the t50 and t90 which is not generally longer. The t50 at 1 Hz was significantly prolonged, but this analogous result must be seen in the context of the markedly different properties between sub-populations of iPSC-CM, in particular between spontaneously beating and non-spontaneously beating cells which on structured constructs have significantly longer tP, t50 and t90 (Fig. 4). The differences between our findings in human iPSC-CM and the published literature on NRVM may be due to inter-species differences or differences in the maturity of neonatal and “embryonic like” cells. This is evident from our provisional experiments with NRVM (Supplemental Fig. 2), which are completely concordant with the published literature showing reduced tP, t50 and t90 at most frequencies. This effect is less evident at 2 Hz where the Ca^2+^ extrusion was not sufficiently developed for it to return to baseline between transients. Finally in our experiments with NRVM there was no significant difference in amplitude between structured and unstructured constructs unlike in iPSC-CM.

Gene expression data did not show difference between structured and unstructured cultures. Even on microgrooved PDMS substrates iPSC-CM continue to express a globally immature phenotype. This suggests that other mechanisms which were not screened here or post translation modifications may be involved in the effects observed. An important caveat is that the summation of gene expression in all cells in a dish may not be representative of the gene expression of individual iPSC-CM in which Ca^2+^ were measured and more sophisticated single cell genetic sequencing techniques should be employed to address these points [37].

It is not clear if ultrastructural reorganisation influences Ca^2+^ handling or whether changes in Ca^2+^ handling are independent. Several studies suggest that aligned cells generate greater force [16,21], and we hypothesise that load may have a role in promoting cellular maturation. This may be supported by our finding that iPSC-CM beating in culture on structured tissue culture substrates had significantly reduced tP, t50 and t90, but cells beating on unstructured constructs did not. Spontaneously beating and non-spontaneously beating iPSC-CM were aligned on structured constructs; however, the fact that spontaneously beating iPSC-CM on structured constructs had reduced tP, t50 and t90 suggests that load may be important. Although spontaneously beating cells may have different physiological properties for other reasons (e.g. a different sub-population with different properties), the fact that spontaneously beating cells on unstructured constructs were not different from non-beating cells suggests that anisotropic load in particular may have an important effect on Ca^2+^ cycling (Fig. 4). This is supported by the rapid changes in cardiomyocyte morphology, Ca^2+^ cycling, and electrophysiology following birth, and evidence from experimental models in NRVM in which stretch has been shown to have a direct effect on Cx43 expression, cell coupling, ion channel activity, and action potential duration [38].

## 5 Conclusion

This study shows that structured tissue culture substrates affects Ca^2+^ cycling and structural properties in cultured human iPSC-CM. This model may be the first step to obtain maturation of iPSC-CM. Further construct development is needed, both to fully interrogate the complex interaction between structure, function and environment and in order to facilitate wider application of iPSC-CM as disease models.

## Figures and Tables

**Table 1:** Summary of PCR primers used in gene expression analysis.

Gene	Encoding	Primer sequence
		(F)orward (5′–3′)
		(R)everse (5′–3′)
ACTB	Beta actin (*β-actin*)	(F)CCAACCGCGAGAAGATGA
		(R)CCAGAGGCGTACAGGGATAG
AMPH2	Bridging integrator 1 (*BIN1*)	(F)ACGGGAGCAACACCTTCA
		(R)GCCGCGAAAACAGTTTACTT
ATP2A2	Sarco/endoplasmic reticulum Ca^2+^-ATPase (*SERCA2*)	(F)AACGTCGGGGAAGTTGTCT
		(R)GAATCAAAGCCTCGGGAAAT
CACNA1C	L-type voltage-dependent Ca^2+^ channel (*Ca*_*v*_*1.2*), alpha 1C subunit	(F)TGACATCGAGGGAGAAAACT
		(R)ACATTAGACTTGACTGCGGC
CACNA1G	T-type voltage-dependent Ca^2+^ channel (*Ca*_*v*_*3.1*), alpha 1G subunit	(F)TGCTCTTCAATTTGCTGGTC
		(R)TCTTCCCGTTTGCTGATTTC
CALR	Calreticulin	(F)CTATGATAACTTTGGCGTGCTG
		(R)CTCCTCAGCGTATGCCTCAT
CASQ2	Calsequestrin 2	(F)GAGTTTGATGGCGAGTTTGC
		(R)TTGCTGCTGATGATCTCCAC
CAV3	Caveolin 3	(F)GAGGCCCAGATCGTCAAG
		(R)TCACGTCTTCAAAATCCACCT
GAPDH	Glyceraldehyde-3-phosphate dehydrogenase (*GAPDH*)	(F)GTC AGT GGT GGA CCT GAC CT
		(R)CCC TGT TGC TGT AGC CAA AT
GATA4	GATA binding protein 4 (*GATA4*)	(F)GGAAGCCCAAGAACCTGAAT
		(R)GTTGCTGGAGTTGCTGGAA
ITPR2	Inositol 1,4,5-trisphosphate receptor, type 2 (*IP3R*)	(F)CCTACTCCAAAACTGCACAGG
		(R)GTCTGACATTGATATCCCCATCT
JPH2	Junctophilin 2	(F)AACATTGCTCGCACTTTGG
		(R)GCTTCTGATATTCCGGACCTG
MYH6	Myosin heavy chain 6 (*α-MHC*)	(F)CTCAAGCTCATGGCCACTCT
		(R)GCCTCCTTTGCTTTTACCACT
MYH7	Myosin heavy chain 7 (*β-MHC*)	(F)ACACCCTGACTAAGGCCAAA
		(R)TCCAGGGATCCTTCCAGAT
MYL2	Ventricular myosin light chain 2 (*MLC2v*)	(F)ACA TCA TCA CCC ACG GAG AAG AGA
		(R)ATT GGA ACA TGG CCT CTG GAT GGA
NKX2-5	Homeobox protein Nkx-2.5 (*Nkx-2.5*)	(F)ACC TCA ACA GCT CCC TGA CTC T
		(R)ATA ATC GCC GCC ACA AAC TCT CC
NPPA	Natriuretic peptide A (*ANF*)	(F)CAGGATGGACAGGATTGGAG
		(R)TCCTCCCTGGCTGTTATCTTC
PLN	Phospholamban	(F)TGATGATCACAGCTGCCAAG
		(R)TGAGCGAGTGAGGTATTGGA
PPIG	Peptidylprolyl isomerase G (*Cyclophilin G*)	(F)CTTGTCAATGGCCAACAGAGG
		(R)GCCCATCTAAATGAGGAGTTGGT
RN18S	18S ribosomal RNA (*18S*)	(F)GCAATTATTCCCCATGAACG
		(R)GGGACTTAATCAACGCAAGC
RYR2	Ryanodine receptor 2 (*RyR*)	(F)CTGCGCCATTCCTATAGTGG
		(R)AGTTGAAGACCGGGAGGTG
SLC8A1	Solute carrier family 8 (Na^+^/Ca^2+^ exchanger), member 1 (*NCX*)	(F)GGTTGGGACTAACAGCTGGA
		(R)CCACATTCATCGTCGTCATC
TCAP	Titin-cap (*Telethonin*)	(F)GGCAGAATGGAAGGATCTGA
		(R) TCTCATGTCTCTGGGTGTCCT
TNNT2	Troponin T type 2 (Cardiac) (*cTnT*)	(F)TTC ACC AAA GAT CTG CTC CTC GCT
		(R)TTA TTA CTG GTG TGG AGT GGG TGT GG
TRDN	Triadin	(F)ACATATTTGTCCATGGGGATTT
		(R)TGGAAGCTTGTTCTGTCGGTA
