# Supplementary material for: The effect of microgrooved culture substrates on calcium cycling of cardiac myocytes derived from human induced pluripotent stem cells
Source: Biomaterials. 2013 Mar;34(10):2399–411. doi: 10.1016/j.biomaterials.2012.11.055 (PMC3605579; doi:10.1016/j.biomaterials.2012.11.055)
Supplement: Supplementary file 1 [file mmc1.pdf]

Supplemental material

Microgrooved Culture Substrates affect Calcium Cycling of Cardiac Myocytes Derived from Human Induced Pluripotent Stem Cells

Rao et al., 2012

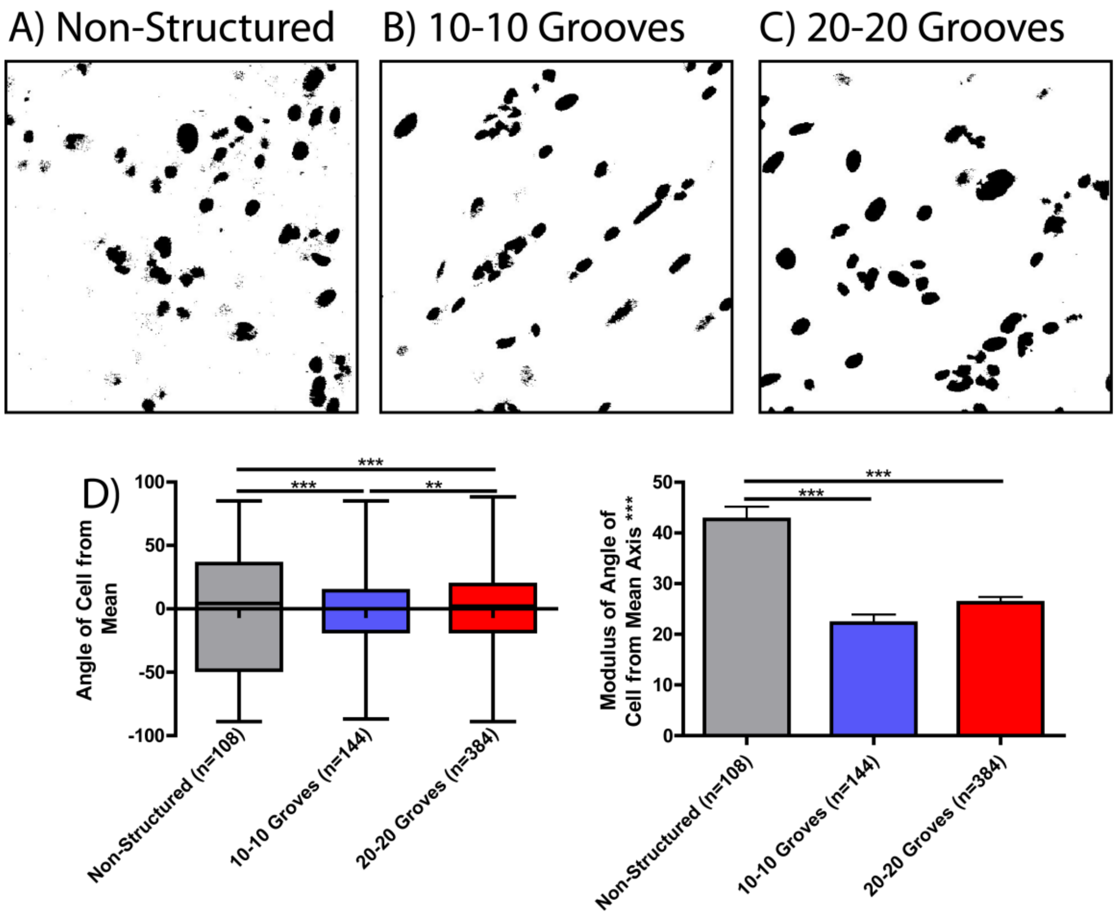

Supplemental Figure 1. Quantification of cell alignment of NRVM on microgrooved PDMS constructs. Immunohistochemistry DAPI images are converted into binary images (see representative examples A-C), alignment is quantified by measuring variation of the long-axis of each nucleus from the mean long-axis.

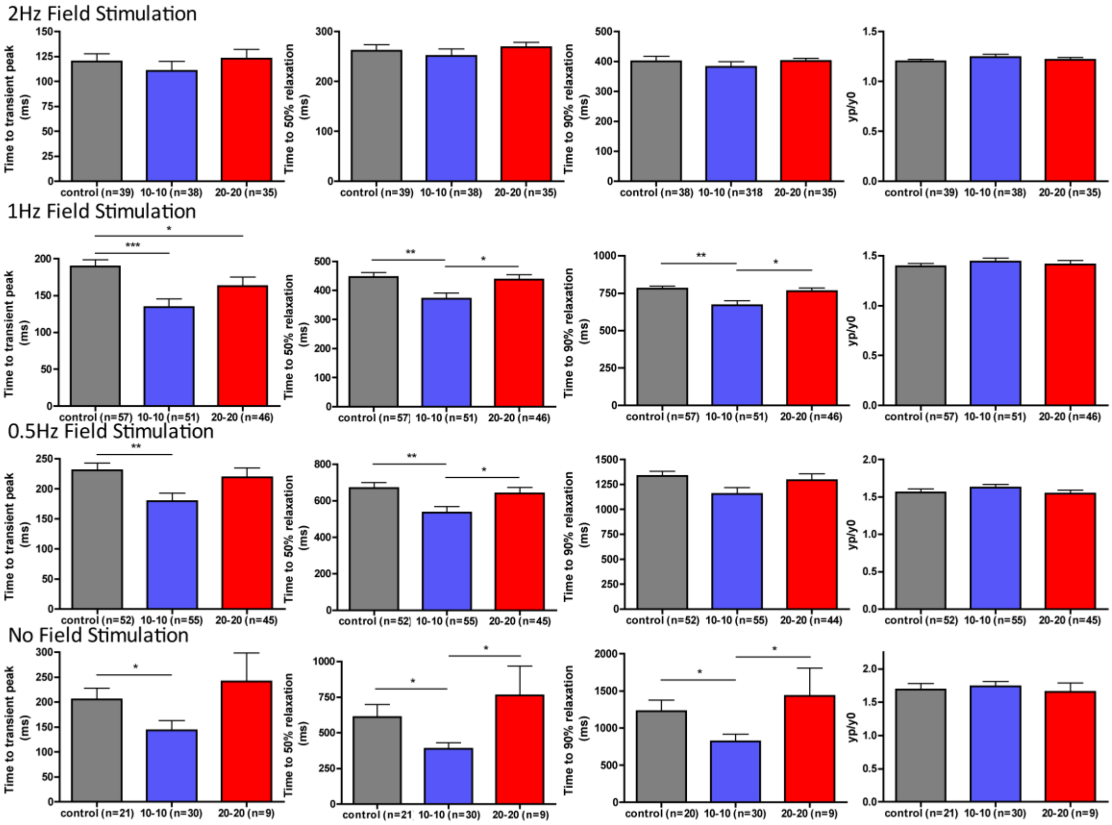

Supplemental Figure 2. Time to Ca^{2+} transient peak (tP), 50% decay (t50), 90% decay (t90), and amplitude of fluorescence (fp/f0) of NRVM cultured on unstructured PDMS, 10-10 and 20-20 grooved constructs field-stimulated at 2Hz, 1Hz, 0.5Hz, and beating spontaneously

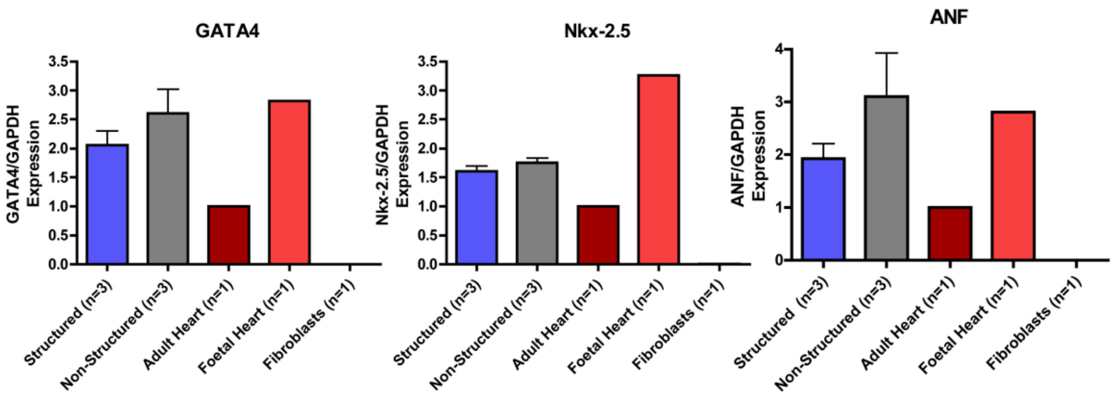

Supplemental Figure 3. Comparison of genetic markers of differentiation (GATA4, NKX2.5, and ANF) when normalized to GAPDH and expressed relative to adult heart tissue in iPSC-CM cultured on structured and non-structured PDMS, fibroblasts, adult heart and foetal heart tissue.

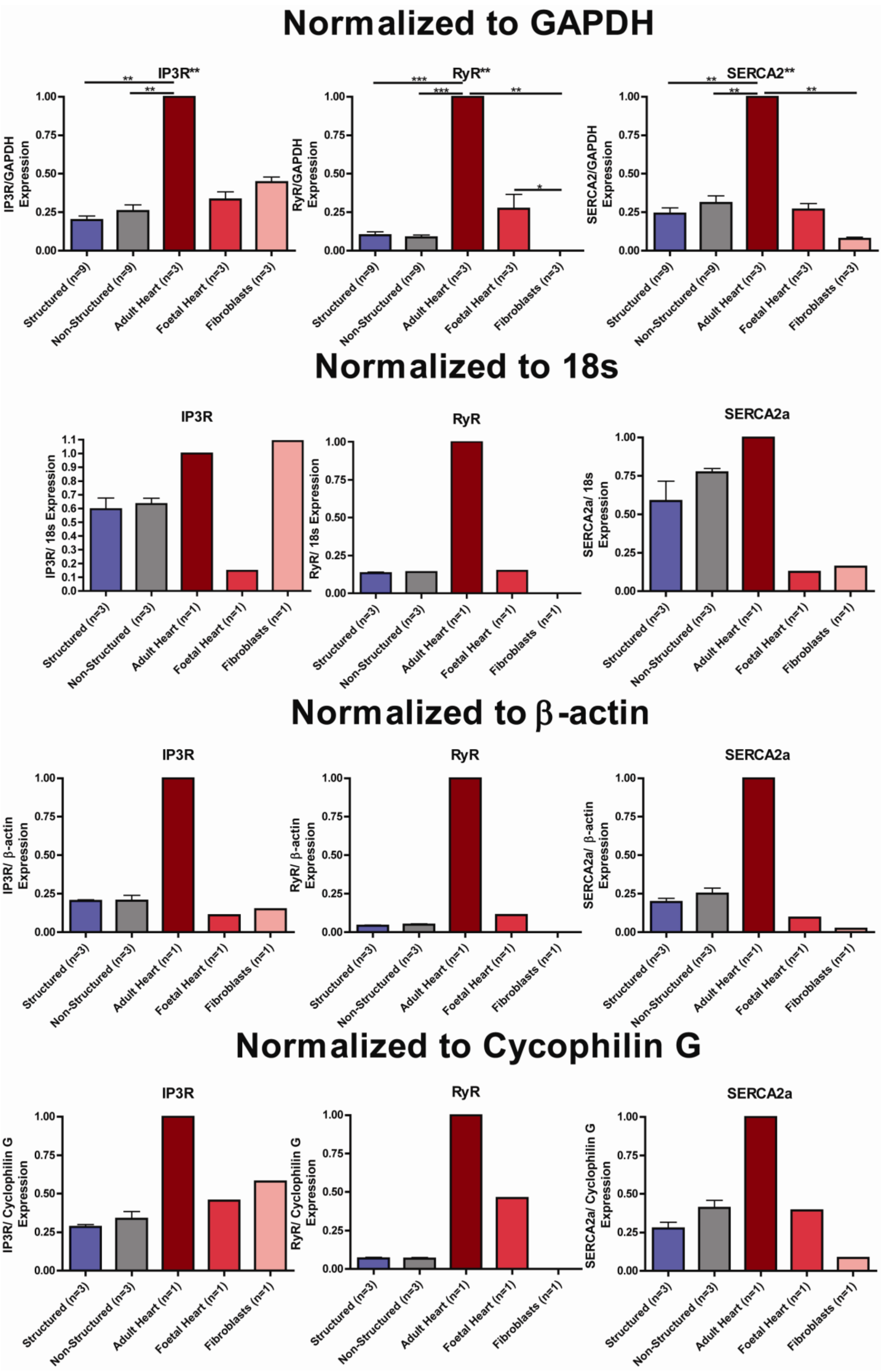

Normalized to GAPDH
IP3R**
IP3R/GAPDH Expression
RyR**
RyR/GAPDH Expression
SERCA2**
SERCA2/GAPDH Expression
Structured (n=9)
Non-Structured (n=9)
Adult Heart (n=3)
Foetal Heart (n=3)
Fibroblasts (n=3)
Normalized to 18s
IP3R
IP3R/ 18s Expression
RyR
RyR/ 18s Expression
SERCA2a
SERCA2a/ 18s Expression
Structured (n=3)
Non-Structured (n=3)
Adult Heart (n=1)
Foetal Heart (n=1)
Fibroblasts (n=1)
Normalized to β-actin
IP3R
IP3R/ β-actin Expression
RyR
RyR/ β-actin Expression
SERCA2a
SERCA2a/ β-actin Expression
Normalized to Cycophilin G
IP3R
IP3R/ Cyclophilin G Expression
RyR
RyR/ Cyclophilin G Expression
SERCA2a
SERCA2a/ Cyclophilin G Expression

Supplemental Figure 4. Comparison of IP3R, RyR and SERCA2a expression when normalized to GAPDH, 18s ribosomal RNA, Cyclophilin G, and β-actin, and expressed relative to adult heart tissue in iPSC-CM cultured on structured and non-structured PDMS, fibroblasts, adult heart and foetal heart tissue.
